# What makes Chinese adolescents “trapped” in severe mental illness? An interactionist perspective on self and identity

**DOI:** 10.1080/17482631.2023.2250093

**Published:** 2023-08-31

**Authors:** Yihan Wu, Marcus Yu Lung Chiu, Weiyun Wu, Sijia Han, Jing Wang

**Affiliations:** a School of Social Development, Nanjing Normal University, Nanjing, China; b Department of Social and Behavioural Sciences, City University of Hong Kong, Hong Kong, China; c School of Health and Wellbeing, University of Bolton, Bolton, UK; d Centre of Mental Health & Society, Bangor University, Wales, UK; e Felizberta Lo Padilla Tong School of Social Sciences, Caritas Institute of Higher Education, Hong Kong; f Affiliated Jianhu Hospital of Nantong University Xinglin College, Yancheng, China

**Keywords:** Chinese, adolescents, severe mental illness, self, identity, constructivist grounded theory

## Abstract

The aim of this study was to explore the self and identity perspectives among Chinese adolescents with severe mental illness (SMI), with a focus on their illness experience and subjective meaning of a formal diagnosis. Thirty-one Chinese adolescents were interviewed and the interview data were analysed strictly according to principles suggested by the constructivist grounded theory approach. Five theoretical codes emerged in this study, including changes of personal values and beliefs, accumulated persistent developmental challenges and personal stresses, ineffective coping strategies and development, symptoms and development of mental illness, and changed perceptions and understandings of self. A proposed model of “The dynamic interactions of Chinese adolescents’ identity and mental illness”, was constructed and visualized. The results revealed that adolescents’ identity formation is a fluctuating and non-linear process, but tends to be predominantly negative. The negative self, as informed by long-term ineffective coping with accumulated persistent developmental challenges and stressful events, develops towards a more serious status of negative identity and contributes to relapse symptoms, although this impact occurs variably with perceived personal characteristics. Besides, some participants who had achieved a state of “Buddha-like numbness” made a conscious decision to live a seemingly normal life while coexisting with their illness. The study also highlighted the positive aspects of identity formation that can arise from the experience of illness, including an enhanced sense of realism and increased empathy. Our findings will imply much the need for person-centred treatment plan and services that take into account of individual situations.

## Introduction

Severe mental illness (SMI) is characterized by severe functional impairments. The three most common SMI are bipolar disorder, schizophrenia, and major depressive disorder (Grotevant, 1987). These conditions are typically associated with significantly impaired social functioning. Globally, the prevalence rate of SMI among people with mental health problems ranges from 1% to 4% (Acero et al., 2017). In China, there has been a worrisome increase in the prevalence rate of mental health issues among school children, reaching 17.5% by 2021 (Li et al., 2022). Consequently, the growing prevalence rate of adolescent mental health issues poses a new challenge for existing school-based mental health work and services (Zeng et al., 2020).

Adolescents with SMI face not only the typical psychosocial developmental tasks, such as identity crisis (Erikson, 1968), but also additional challenges related to their illness. These challenges include academic difficulties, medication management, mental health treatment, lifestyle adjustments, disclosure, and stigma management. These additional crises can further hinder their identity formation, social stability, recovery from illness, and successful reintegration into school, family, and society (Acero et al., 2017; Grotevant, 1987; Jaiswal et al., 2020; Kerr et al., 2019; Van Bulck et al., 2019; Wu, 2022). Therefore, there is a pressing need for mental health support for school children with mental health problems to create a nurturing and therapeutic environment for their healthy development.

The concept of identity and identity crisis was pioneered by Erikson (1968) within a psychoanalytic framework. According to Erikson (1968), an identity crisis is a key element of the structure of identity. It arises when adolescents have to confront conflicts within themselves, presenting varying selves under different circumstances. These circumstances lead to an identity crisis that necessitates an immediate decision about oneself. In this study, the term “identity issue” is used to refer to the identity crisis experienced by adolescents with SMI. Although various researchers have adopted Erikson’s concepts to explore the process of identity formation, their models or empirical research does not fully describe the actual life experiences or underlying dynamic processes of adolescent identity formation and status (Bogaerts et al., 2021; Hihara et al., 2019; Lind et al., 2019; Shiner et al., 2021). Other researchers have focused more on illness identification, social identity, self-image, or an illness identity that encompasses others’ perceptions, evaluations, or responses regarding their illness (Charmaz, 2014; Oris et al., 2018; Ottewell, 2018; Pérez-Corrales et al., 2019; Postmes et al., 2019; Van Bulck et al., 2019; Wicks et al., 2019; Yanos et al., 2020).

Despite these varied perspectives, all these studies emphasize the strong connection between identity and self-awareness. Erikson posits that identity is not limited to a partial part of self-awareness but takes into account both social and individual perspectives. Identity involves representations and feelings and cannot be reduced to a simple cognitive system (Erikson, 1968; Karwowski & Kaufman, 2017). Furthermore, Erikson views identity as a continuous, lifelong developmental process rather than a static status in the present (Erikson, 1968). Consequently, in this study, identity refers to the ongoing self-determination process, integrating the internal self and interactions with multiple social roles. It encompasses an understanding of who one wants to be, who one is or has become, how one perceives others’ thoughts, the perspective of a mental illness diagnosis, and the dynamic nature of adolescent identity development.

Outside the medical research field, adolescent mental illness, particularly those related to emotions, remains controversial. Some researchers suggests that SMI exists because society and concerned institutions define it as such (Fitzgerald et al., 2016; Furr, 2022; Horwitz, 2020). In other words, adolescents produce or exhibit ideas or behaviours that are classified as SMI and its symptoms within certain situations and cultural contexts. Their subjective experiences, along with the SMI symptoms, are evaluated and assessed accordingly. SMI is primarily a diagnostic and clinical term lacking definitive explanations and assessments, without clear evidence. Other fields offer alternative ways to explain so-called SMI and its symptoms (Furr, 2022). However, the crucial question to address beyond these explanations and assessments is how we can understand adolescents’ life stories and support their recovery from SMI, enabling the formation of positive identities beyond their struggles. Additionally, the treatment of mental illness heavily relies on the availability, accessibility, and affordability of professional expertise from psychiatrists and other mental health professionals. Data-based diagnostic and analysis methods, such as imaging evidence and biological indicators, remain limited (Zheng & Zheng, 2015). Therefore, from a non-medical perspective, it is crucial not to overlook the impact of individual differences and the experiences of individuals with mental illness (Horwitz, 2020; Yanos et al., 2020).

Chinese adolescents represent a unique population facing specific challenges related to mental health (Li et al., 2022; Wu, 2022; Zeng et al., 2020; Zheng & Zheng, 2015). Extensive literature review reveals that cultural and societal factors significantly influence the experiences and outcomes of SMI in this group (Huang et al., 2017; Li et al., 2022; McAneney et al., 2015; Zeng et al., 2020). Collectivist values, familial dynamics, educational pressures, and societal expectations prevalent in Chinese culture all shape the manifestation, diagnosis (Ho & Goh, 2017), and treatment of mental illnesses (Huang et al., 2017). Understanding these cultural nuances is essential to develop effective interventions and support systems (Li et al., 2022). Moreover, recent statistics indicate a concerning rise in the prevalence of SMI among Chinese adolescents (Li et al., 2022; Zheng & Zheng, 2015). Therefore, it is imperative to address knowledge gaps concerning this population and explore strategies for providing tailored and culturally sensitive care. By delineating the distinct characteristics and considering cultural considerations, this study aims to contribute to the existing literature and guide future research and clinical practice in addressing the mental health needs of Chinese adolescents with SMI. The research questions to be answered include: What is the identity of Chinese adolescents with SMI? How do Chinese adolescents with SMI experience their illness condition? What factors contribute to SMI in Chinese adolescents?

## Methods

### Study design

Constructivist grounded theory was chosen as research methodology of the current study due to its suitability for capturing the complex and dynamic nature of the phenomenon under investigation. Constructivist grounded theory aligns with the constructivist paradigm, which acknowledges that knowledge is actively constructed through social interactions and subjective interpretations (Charmaz, 2014). Compare to Glaser and Strauss’s grounded theory, constructivist grounded theory places greater emphasis on the importance of context, subjectivity, and the co-creation of meaning between researchers and participants (Charmaz, 2014), allowing for a deeper portrayal of the complexity and characteristics of Chinese adolescents with SMI in their specific socio-cultural contexts.

## Participants and sampling

This study recruited participants through three mental hospitals situated in the Zhejiang province of China with purposeful and theoretical sampling methods (Charmaz, 2014). Purposive sampling method was chosen at an initial stage, to select participants who fitted the purposes of this study through selection criteria in each of the three hospitals. Theoretical sampling method was employed until some analytical and abstract codes had been developed (Charmaz, 2014). During the theoretical sampling phase, a deliberate effort was made to recruit a higher number of male adolescents diagnosed with schizophrenia. This strategic decision aimed to explore the relationship between different genders, SMI, and identity development, with the ultimate goal of achieving theoretical saturations and deriving a more compelling theory. In total, thirty-one adolescents diagnosed with SMI participated in the study, all of whom were currently receiving medical treatment and were either in a stable or clinical recovery phase.

## Ethical considerations

Ethical approval for this study was obtained from College Human Subjects Ethics Sub-Committee of researchers’ institution (No. 55295357). Following that, all research participants willingly agreed to take part in the study by signing the adolescent informed consent form, and their parents signed the parent/guardian informed consent forms. Participants were allowed to refuse to answer personal questions during the interview and to withdraw from the study at any moment without any forms of punishment.

## Data collection

Data were collected through individual in-depth interviews from 21 July 2020 to 12 September 2020. Each interview lasted for a period of 68–120 minutes in private hospital rooms. An interview guidance was developed and used for interviews to guide the conversation and elicit relevant information. The main topics of interview guidance were included self-introduction and growth stories, family, life in school, experience at the mental health institutions, and plans for the future. All these interview topics were considered relevant to the research questions of the current study. At the end of each interview, each interviewee was given 50 RMB as a token fee for their time and effort, out of respect.

## Characteristics of participants

Table I in the following provides a concise overview of the demographic characteristics of the participants. Of the total 31 participants, Q Hospital contributed 10 participants, while K Hospital and S Hospital each provided 10 and 11 participants, respectively. The participants encompassed a spectrum of psychiatric diagnoses: 11 were diagnosed with bipolar disorder, 10 with major depression, and 10 with schizophrenia. In terms of gender distribution, the group consisted of 15 male and 16 female adolescents. Their ages ranged from 13 to 19 years, with a mean age of 16.35 years, thus encompassing the entirety of the adolescent stage, including early, middle, and late adolescence. The duration of illness among the participants varied from 2 to 7 years, with an average duration of 4.03 years.

**Table I.** Demographic characteristics of participants.

Demographic characteristic	Number (n)	Proportion
Hospital		
K Hospital	10	32.26%
Q Hospital	10	32.26%
S Hospital	11	35.48%
Gender		
Female	16	51.61%
Male	15	48.39%
Age (year)		
10–14 (early adolescence)	9	29.03%
15–17 (middle adolescence)	10	32.25%
18–19 (late adolescence)	12	38.70%
Diagnosis		
Major depressive disorder	10	32.26%
Bipolar disorder	11	35.48%
Schizophrenia	10	32.26%
Duration of illness (year)		
2–3	12	38.70%
4–5	9	29.03%
6–7	10	32.26%

## Data analysis

Each audio recording was transcribed within 24 hours. The textual materials were the transcripts of the in-depth interviews, the research diary, and the interview notebook. It should be noted that because the participants of this study were Chinese adolescents, all the interviews were conducted in Chinese language. With respect to the original language used in the interviews, all the data was also analysed in Chinese, and only the supporting excerpts were translated into English in the description and presentation stage. The translation has been double-checked by professional translators and peer-reviewed by co-researchers.

Initial and focused coding were conducted with the data with strategies from the constructivist grounded theory approach suggested by Charmaz (2014). The constant comparative method was continuously used to make comparisons between memos, codes, and the data, grouping and integrating certain initial codes into focused codes which would have a higher analytical value, occurred more frequently, or were closely relevant to the research questions.

## Results

The core category included 5 focused codes, namely, changes of personal values and beliefs, accumulated persistent developmental challenges and personal stresses, ineffective coping strategies and development, symptoms and development of mental illness, and changed perceptions and understandings of self. Through a process of theoretical sampling mainly focused on male participants with schizophrenia and major depressive disorder, the theoretical properties of the concepts became concrete and the theoretical connections between the concepts became clearer. Hence, the core category and its boundary were redrawn, and its purpose and influencing role were pinpointed.

## Changes of personal values and beliefs

Adolescents’ personal values and beliefs were constructed in the social context while influenced by many external and internal elements. There were 11 personal values and beliefs that the study participants mentioned most frequently in the interviews, which were closely related to their identity formation. Firstly, it appeared that their attitudes and understandings of the life, attitudes, and views on others’ views on themselves, attitudes and views on family or parents, attitudes and views on peers or friends, and attitudes and views on education, contributed to their concepts and evaluations of themselves as an individual self. For example, P26 used to be trapped in a thought of trying to be normal or not, in the times of being seriously ill, but his attitude and understanding of life gradually changed during the clinical recovery phase:

P26 (major depression, 6 years): I was constantly in a dilemma, wondering whether people like us should put on masks to pretend to be normal or live as what we are. When I think about it now, I was quite silly at that time. In fact, as long as we could follow our hearts, we would live comfortably and leave that sort of question behind.

Knowledge, attitudes and understandings of mental illness, attitudes and views on the illness experience, attitudes and views on mental health institutions, attitudes and views on mental health staff, understandings and attitudes on the illness treatment and recovery, and attitudes and views on others’ views on mental illness contributed to their concepts and evaluations of SMI and being a person with SMI. Any changes in their development would result in a change of their values and beliefs. As P27 shared that, he changed the values and beliefs about the SMI and person with SMI after being diagnosed with schizophrenia:

P27 (schizophrenia, 2 years): … I lived with my grandfather. There was a beggar (or vagrant) in this neighbourhood. All neighbours claimed him to be mentally ill and hopeless, but I just felt sorry for him. I did not understand why he lingered. Wouldn’t it be better for him to stay indoors without other’s judgement? Now I think about it, I was bitterly disappointed at them, and more at the world. As the saying goes, Rome was not built in a day, and we were not to get sick in a day. It is the world that is sick, but it is we who are falling apart.

Adolescents’ personal values and beliefs were influenced by and also influenced adolescent development, and were thought to be a basis of forming the self and identity. Suffering from SMI was thought to be a frightening and difficult experience, making all the adolescents with SMI pessimistic about their life and self, and making their sense of self into more seriously negative statuses. As P28 shared his values and beliefs in the following:

P28 (bipolar disorder, 4 years): … my mother told me I was a nice kid, but I just did not think so. I did not think I was nice and kind, I could not feel the love and kindness by others either … So, I am insensitive to my goodness. I become irritable, violent, prejudiced, and dark. I feel like I am targeted by those around me. I am not who I used to be.

With the development of SMI, the chronic illness experience gave participants more realistic and empathetic values and beliefs, allowing them to accept the illness and re-form their identity. As P12 put it:

P12 (schizophrenia, 3 years): I am starting to see relapse in a different light. Schizophrenia is at least treatable and manageable. I think it is good to hear such disease can be medically controlled. I think it is not the illness per se, but the problems or symptoms that come with it that keep me from moving forward with my life. Compared to those who cannot be treated, whether it is a relapse or not, we are a bit better.

Despite the fact that SMI experiences could bring the feature of realism and empathy, these adolescents also realized the challenging nature of dealing with the many additional stresses and challenges caused by SMI. The SMI experience was considered a chance to rethink the self and reform identity, but also a barrier to a good life. These adolescents were still trapped in pessimistic values and beliefs, as stated by P26 and P25:

P26 (major depressive disorder, 6 years): We cannot change our past. It is more like playing a game. Once it started, we can never go back. Though we cannot change the bad things in the past, we could learn to accept what it is and make our lives a bit easier. For me, being sick is a process of living towards death and self-acceptance.

P25 (bipolar disorder, 6 years): I am now very regretful that I did not continue my studies. If things could start over, I would compel myself to hold it a little longer. If I had given it a shot, all the regrets afterwards would have a different meaning.

The code of “changes of personal values and beliefs” in this study links to the rest of the codes by indicating the influence of personal values and beliefs on the self and identity development, particularly in the context of mental illness. The study participants mentioned various personal values and beliefs that were closely related to their identity formation and self-concept, such as attitudes towards life, family, peers, education, mental illness, and recovery. These personal values and beliefs were influenced by both internal and external factors, including their own experiences with mental illness, interactions with others, and societal views on mental health. Changes in these values and beliefs were observed to occur over time and were intricately connected to personal growth and the ever-changing living environment of the adolescents. The changes in personal values and beliefs also had implications for the participants’ experiences of living with a mental illness. For some, the experience of mental illness led to negative self-perceptions and pessimistic values and beliefs about themselves and their future. Others, however, found that their illness experience provided them with a more realistic and empathetic perspective, allowing them to accept their illness and re-evaluate their sense of self and re-form their identity. Furthermore, the code of “changes of personal values and beliefs” is seen as a link to other codes related to the participants’ accumulated persistent developmental challenges, personal stresses, and ineffective coping strategies. The changes in personal values and beliefs influenced how the participants perceived and responded to these challenges, which in turn could impact their overall development and well-being. Overall, the code of “changes of personal values and beliefs” serves as a significant factor in understanding the complex interplay between mental illness, identity formation, and adolescent development. It highlights the dynamic nature of personal growth and the potential impact of shifting values and beliefs on adolescents’ experiences and outcomes.

## Accumulated persistent developmental challenges and personal stresses

In the process of growing up, adolescents with SMI have encountered a total of 11 developmental challenges and personal stresses closely related to their identity formation, as shown in Table II.

**Table II.** Developmental challenges and personal stresses.

Developmental challenges and personal stresses	
1	Difficulties in establishing a trusting doctor-patient relationship
2	Difficulties in establishing close friendship or romantic relationships
3	Difficulties in establishing mutually dependent parent-child relationships
4	The possibility of disease recurrence
5	The side effects of treatment
6	Expensive medical expenses
7	Fear of discrimination or prejudice from others
8	Academic pressure
9	Competition with peers
10	Interruption in education
11	Resuming studies

For example, as shared by P2, she had a difficult time with her counsellor, and found it difficult to establish a trustful doctor-patient relationship. The negative experience with her counsellor triggered a cascade of negative emotions, leading to the feeling overwhelmed. These emotional responses can be categorized under the code “symptoms and development of mental illnesses”. Besides, her avoidance of counselling despite acknowledging its necessity showcases the internal struggle she faces. This struggle reflects a changing perception of herself, where she becomes unsure of her ability to benefit from counselling or manage her major depressive disorder effectively. This change in self-perception aligns with the code “changed perceptions and understandings of self”:

P2 (major depressive disorder, 4 years): I began to doubt myself and became overwhelmed. But reason told me, “I still need counselling”, but inside of it I kept avoiding it. And my stress intensified at the thought of counselling.

Adolescents also encountered interpersonal difficulties both at school and within their families. These challenges had a negative impact on their self-perception and led to certain symptoms of mental distress. For instance, P7 shared that they felt isolated from others and lacked the energy to engage with peers or maintain social connections in school, especially in the first year of high school. These experiences highlight the struggles in forming and maintaining meaningful relationships, which can contribute to feelings of inadequacy and a negative self-image. Similarly, P4 mentioned that an unsupportive family environment discouraged him from socializing. This, in turn, contributed to low self-esteem and fear, causing him to isolate himself from others. Additionally, P4 expressed feeling unsafe at home and experiencing bullying outside, which further hindered their ability to form healthy relationships and engage in positive social interactions. These cumulative factors present significant challenges in establishing connections and participating actively in social settings.

P7 (bipolar disorder, 2 years): I struggled to learn when to say and what people like to hear, so that they would feel happy with me. I also kept myself apart from other people. I could control my emotions and be rational. But I was having a really hard time. By my first year of high school, I did not have the energy to get along with people or keep in contact with anyone.

P4 (bipolar disorder, 2 years): Those family chores have discouraged me from socialising ever since I was a child. I was always full of worries because of low self-esteem or fear. I kept myself away from other people and naturally I did not have many friends. I did not feel safe enough at home and I was often bullied outside.

One developmental challenge or personal stress after another followed the changes of living environment and personal growth experience, bringing accumulated and ongoing pressure. These developmental challenges and personal stresses gradually accumulated alongside the challenges of adolescent development and moved from a state of latency to an explosion. As indicated by P11:

P11 (major depressive disorder, 5 years): I was selected for the Maths Olympiad class, but my grades were only in the lower middle of the class. At that time, I was such an extreme perfectionist that the decline in my grades was too much for me to take and I completely lost my mind. I was under tremendous pressure to study, and because of the exams, I felt very anxious and could not control my anxiety. It became worse on the eve of the exams. I felt like I was going to be mentally dismantled in the next second during the exams. Stress and worries piled up and I slowly crumbled. During the period of school exams, I was very pessimistic day by day, crying and worrying that the world would soon collapse.

With the SMI development, adolescents realized that they had additional challenges and stresses caused by SMI. These included the stress due to possible relapse; stress caused by side effects of the medicine; stress caused by the high cost of treatment; stress caused by fear of discrimination or prejudice by others; stress caused by study and education; stress caused by competition with peers; stress caused by schooling suspension; stress from returning to school. These additional challenges and stresses created a more negative self and stimulated certain SMI symptoms.

For example, P7 found, when he chose to receive medical treatment and professional intervention, that such experiences brought additional stresses which he had to face and cope with, and he further caused negative feelings such as “exhausted”. Similarly, P9 found the uncertainty of the future because of the potential relapse of SMI, the “lifetime relapse” stopped her normal life, and made her more pessimistic about life and self. For example, P7 found, when he chose to receive medical treatment and professional intervention, that such experiences brought additional stresses which he had to face and cope with, and that they further caused negative feelings such as “exhausted”.

## Ineffective coping strategies and development

When adolescents with SMI in this study were faced with the accumulated persistent challenges and stresses as described above, these adolescents seemed to develop five coping strategies. The first was coded as “active help-seeking”. It reflected the adolescents’ initiative in seeking help from professionals or close personal contacts. Through actively seeking helps, adolescents could express their negative emotions and cope well with their negative thoughts, but it could not work in the long term.

P4 (bipolar disorder, 2 years): The first time I sat in the school counselling room, I felt stressed out because of academic problems and conflicts with my classmates. I went to seek help and made an appointment, and for an hour the teacher (counsellor) barely spoke. I had so much to say, gushing and crying for a moment. The counsellor did not say anything until the last minute. He told me I was just thinking too much or something to comfort me, like everyone else. This is the main reason why I never went to the hospital because I knew it would be useless and that no one would understand me.

“Passive obedience (被动地服从)” and “voluntary obedience (negative) (消极地顺从)” are two coping strategies for identity crisis, SMI symptoms, developmental challenges, and personal stresses. Passive obedience is when adolescents comply with external demands without expressing their own desires or opinions. Voluntary obedience, in a negative sense, is when adolescents comply without personal ownership or engagement. Despite conforming outwardly, those employing voluntary obedience negatively may feel frustration, resentment, or detachment internally.

P10 (major depressive disorder, 4 years): … When my mother said, “If you didn’t study well, you would become a worthless person, and your life would be just a waste of food” … even though I was wronged and uncomfortable to hear these words, I still accepted it unconsciously… and gradually I was “pushed” to be a perfectionist in that way … I felt miserable and painful when I was not able to meet others’ expectations.

P24 (bipolar disorder, 4 years): … I had been a walking dead, tired of concealing, tired of repression, tired of controlling my energy, so I did everything the doctor told me to do … took medicine … took exercise. I knew I was vulnerable, and I wanted to go back to where I was, but I thought I might collapse at any point …

In addition to the mentioned strategies, the strategies of “active resistance” and “confront with challenges and stresses (pessimistic)” also reflected adolescents’ limited coping resources and ability due to their characteristics and experiences, which resulted in the development of ineffective coping strategies. As shared by P19:

P19 (schizophrenia, 3 years): … later, I was diagnosed with schizophrenia and the doctor told I had to take medicine. I pretty much doubted his diagnosis. I felt like I just came to the hospital for my anxiety. How come it became schizophrenia all of a sudden? … just no way … . The doctor told me to take medicine, but I refused. Why did I have to take medicine anyway? And I thought the doctor misdiagnosed this time … I just thought if I really took this medicine, the medicine for treating schizophrenia, I definitely would be pushed to become a mad person finally …

P10 (major depression, 4 years): I realized that mental illness was also a way to somehow remind and protect me, I stopped to reject it that much, and I also stopped hating myself that much … I thought that “she [myself] was a vulnerable person, also needed to be taken care of. So why pretend to be strong” … So, I slowly asked myself not to compete with peers around me … always told myself, “living for the sake of living … and that’s all”.

The current focused code appeared to be linked with the other focused codes of this category. One challenge after another as well as stress caused SMI symptoms and negative self under the ineffective copings. It further seemed to lead to a pessimistic attitude towards the sense of identity and the real world, as explored in the following sections.

## Symptoms and development of mental illness

The symptoms and development of mental illness of adolescents with SMI includes psychotic symptoms, manic symptoms, depressive symptoms, complication symptoms (self-injury, suicidal thoughts, and behaviours), and disappearance of pathological symptoms (in the clinical recovery phase). For example, P5 recalled and shared a kind of psychotic symptom at the time being seriously ill, whereas P4 recalled and shared some manic symptoms during his manic phase.

The onset of adolescents’ mental illness in this study concentrated in middle school, but there were already accumulated impacts on their emotions, cognitions, physical function, or behaviours before the onset of mental illness appeared. As indicated by P1, she suffered a long-term insomnia before the diagnosis of a major depressive disorder three years ago:

P1 (major depressive disorder, 3 years): I could not sleep at night, inactive to attend classes or concentrate on my homework during the day. I feel numb when reading words. I can read every word, but it takes me a long time to know what the sentence means.

The difficulty of sleeping became much more serious as it developed, gradually damaging her body functions and health, as continued by P1:

P1 (major depressive disorder, 3 years): I could not follow when others talked to me, and I could not concentrate on reading. After that, I had an emotional breakdown and took a week off to stay home. I could not sleep either, staring at the ceiling with my eyes wide open from dark to dawn every night.

The onset of SMI affected not only the parent-child relationship, but also peer and doctor-patient relationships. For example, P22 described his first diagnosis experience, explaining that he showed a lack of insight during the onset of SMI, which not only led to a deterioration in his condition, but also to a distrustful doctor-patient relationship.

Moreover, most adolescents with SMI were suffering from major depressive disorder and had a clear understanding of their own changes, and some with bipolar disorder had a correct understanding and explanation of their own changes; but none of the adolescents with schizophrenia could clearly identify their pre-illness symptoms. Those adolescents who did not have a clear understanding and perception of illness, preferred to explain such changes as deviant behaviour or physical illness. When they are unable to recognize or attribute their symptoms to a mental illness, which contributes to difficulties accepting and coming to terms with their condition, potentially impacting their self-perception and overall identity development. For example:

P3 (major depressive disorder, 4 years): I have suffered from irritable bowel syndrome since kindergarten. When my illness broke out the very first time, the symptoms became very serious, accompanied by nausea, loss of appetite, and chest distress. Who would link this to mental illness? No one, including me … .

## Changed perceptions and understandings of self

Adolescents’ perceptions and understandings of self changed over time. The code “ambiguity (in vivo, 迷茫)”, “confusion (in-vivo, 混乱)” and “denial (in vivo, 否定)” were thought to be statuses of perceptions and understandings of self. Adolescents with SMI gradually developed a perceived sense of negative self-alongside the development of SMI. For example, P2 perceived that she had an ambiguous sense of self when feeling separated from a parent, P21 perceived herself as having a confused sense of self when suffering from repeated misdiagnosis, whereas P22 perceived he had a denied self at the time of onset of schizophrenia:

P2 (major depression, 4 years): … it is hard to define the closeness of my connection with them [my parents]. They [my parents] were both busy with work. My grandparents were both old. There was no choice for them, probably … . But I used to always have an ambiguous sense of self, I used to always think that what were parents? What it was like to be living with parents? What a real home looked like? …

P21 (bipolar disorder, 7 years): … . I am lost in my own world every day and I am always angry with myself and cannot talk to anyone else. I am also very unstable, being awake and being confused for time to time…

P22 (schizophrenia, 7 years): … but when I got sick, I am broken, giving up the struggle, often repeatedly denying myself and suddenly losing control. It seems that illness made me worse … .

These statuses did not develop linearly but exhibited fluctuations over time, going back and forth. For example, P6 recognized she was in “confusion” in respect of her family identity in early childhood due to family conflict, “ … a question always popped up in my mind at that time, ‘why do I have to face up to all this?’”, and became “ambiguity” due to negative interpersonal experience in school, “ … I really questioned whether I was as vulnerable as they thought I was … ”, but returned to “confusion” in herself again during the medical treatment, as shared in the following:

P6 (bipolar disorder, 6 years): … the biggest problem at the time was that I did not know what direction to take with my life. It felt like I knew what I liked but did not know if I could stick with it. And also, I was poorly educated, lacking experience and ability.

Part of the adolescents with SMI (or previously) developed “mental numbness (病态性麻木)”. Unlike psychic numbing, the mental numbness does not exclude the possibility of psychosis, but a psychic numbing refers specifically to an episode of psychosis. The term more of a sense of “dead” self, which was seen as potentially leading to a numbness across time. This numbness was considered morbid, as some adolescents were numb due to SMI symptoms and the onset of SMI, some (previously or currently) refused to accept their illness, some (previously or currently) had no future plans, and, with a feeling of despair or anxiety about the future, some previously had relapses associated with non-compliance of medication.

P26 (major depressive disorder, 6 years): … by then I had learned to control my emotions, but I had also turned myself into a numb person. The love of colour and music was gone, too. I forced myself to do as much exercise as possible every day. I know that that was not me. I hated being numb, which at least protected me from my emotional turmoil though. I thought it was not unfeasible as long as I could keep myself calm and not relapse.

A comparison between the above status and “Buddha-like numbness (佛系麻木/非病态性麻木)” is implicit in this focused code Buddha-like. It appeared that these adolescents had developed a Buddha-like attitude (the term Buddha-like has been widely spread and popularly discussed through the Internet in China. It refers to a peaceful mindset and has nothing to do with Buddhist theology), and a feeling of “missing” past opportunities and losing chances due to the diagnosis of SMI. Unlike being morbidly numb, some of these adolescents accepted their SMI and illness identity, while some chose to numb their life, living in a manner offering comfort to themselves and refusing to plan for the future. They believed, or chose to believe, that such an approach would keep them safe and protect them away rejection. As continued by P26:

P26 (major depressive disorder, 6 years) … I am afraid it is going to be a lifelong dream for me to get back to the way I was before I got sick, able to be energetic and work as hard as I did before. Because of this illness, I have had to live with a lot of unbearably messy consequences when my sanity returns. From the anxiety before to the pessimism now, I found that choosing to be numb and lowering requirements is the right way for me to live. Since my perfectionism exhausts me, I choose to take one step at a time and live rough with less concerns. I am still a sensitive and pessimistic person, anxious about the uncertainty of the future, unjust about reality, and regretful about missing out. But there is also a real sense of relaxation that was not there before.

## The proposed model

**Figure 1.**
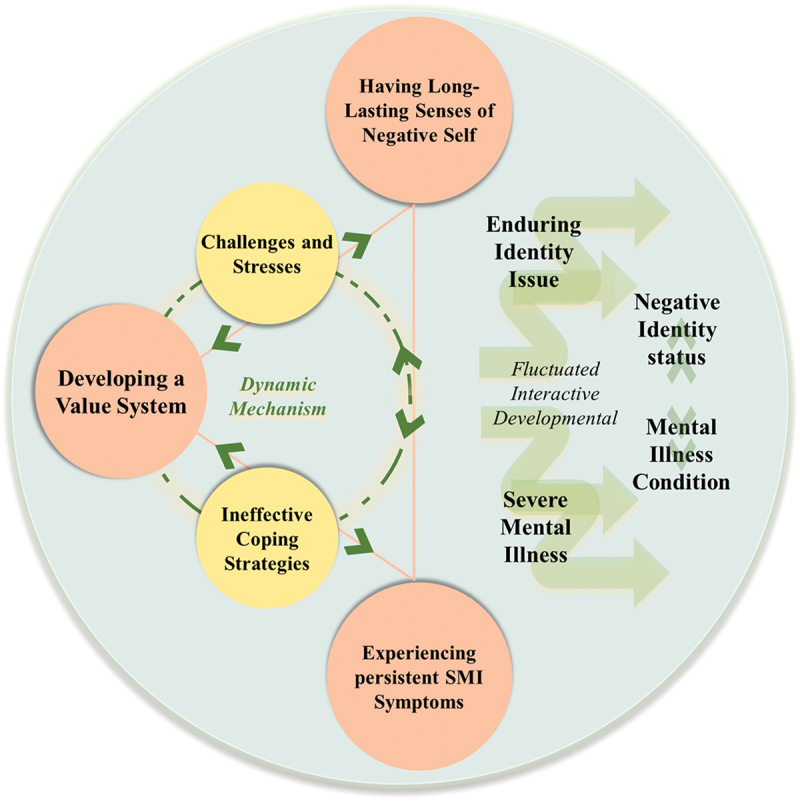
The dynamic interactions of Chinese adolescents’ identity and mental illness.

The proposed model of “The dynamic interactions of Chinese adolescents’ identity and mental illness”, illustrated in Figure 1, visually represents the interactions between codes and their impact on adolescents’ negative identity status. The model demonstrates that this status is not static, but rather undergoes dynamic changes and fluctuations over time.

Specifically, one important aspect of these changes is the influence of adolescents’ value system (i.e., focused code of changes on personal values and beliefs). Adolescents’ value system is shaped and developed through interaction with other codes. As adolescents interact with others and experience new situations, their value system will undergo shifts and modifications. Another crucial dimension in this process is the development of long-lasting negative self-perceptions (i.e., focused code of changed perceptions and understandings of self). How adolescents perceive and understand themselves can change during their developmental journey. It interacts with the dimension of experiencing persistent symptoms of mental illness (i.e., focused code of symptoms and development of mental illness). The symptoms and development of mental illness are intimately connected to identity formation across the time.

Taking a life-course perspective, the dynamic mechanism of development is both ongoing and cyclical. Ineffective coping strategies contribute to this dynamic cycle (i.e., focused code of ineffective coping strategies and development), which in turn contribute to the persistent challenges and stresses and development of ineffective coping strategies (i.e., focused code of accumulated persistent developmental challenges and personal stresses) across the time. It’s crucial to note that this model does not follow a linear understanding of change. Instead, it recognizes the fluctuating nature of identity formation due to developmental interactions. Adolescents’ identities are not fixed but evolve through interactions and are influenced by various factors throughout their developmental journey. In summary, the proposed model visualizes the dynamic and fluctuating nature of adolescents’ negative identity status. It highlights the importance of interactions between codes. This model offers a comprehensive understanding of how these elements intertwine and shape the process of identity formation during adolescence.

## Discussion

All adolescent accounts provided detailed descriptions of their experiences with serious and chronic psychological and mental illnesses. However, some participants who had attained a state of “Buddha-like numbness” described their recovery process as a conscious decision to live a seemingly normal life while coexisting with their illness. They emphasized the notion of actively choosing this restricted and unfortunate situation. The close link between serious mental illness and issues of identity prompted us to explore the positive aspects of identity formation that may arise from the experience of illness, suggesting the presence of positive psychological traits associated with SMI. One positive consequence of the SMI experience is an enhanced sense of realism. It appeared that the experience of SMI helped these adolescents develop a more realistic perspective, particularly in terms of their pessimism. This enabled them to accept their diagnosis and foster a sense of authenticity and self-awareness within their lived reality. This finding aligns with previous research indicating that individuals with depression tend to exhibit a more realistic sense of control and have a better ability to forecast post-treatment life events (Feltham, 2016; Lyons & Carhart-Harris, 2018). Another positive outcome of the SMI experience was increased empathy. Participants who achieved Buddha-like numbness reported that they developed a sense of sympathy towards themselves and accepted their illness and its impact on their lives. Additionally, they expressed empathy towards the suffering of others. Though they may not have had a clear and positive plan for their own future, they expressed a willingness to assist individuals with SMI in the future. This finding is consistent with studies on posttraumatic growth and stress-related growth in mental illness conducted by recent studies (Lee et al., 2022; Ng et al., 2021; Slade et al., 2019), which highlighted changes in relationships and increased sensitivity. Since empathy is fundamentally a response to observing another person’s experiences (Furnham & Sjokvist, 2017), it supports the argument that psychological and mental illness experiences can contribute to improved empathy.

In addition to personal factors, our study also found that culture played a significant role in the experiences of Chinese adolescents with SMI. Although not explicitly discussed by all participants, some descriptions provided by the Chinese adolescents were influenced by certain aspects of Chinese cultural values. For instance, Participant 10 shared an example of how their mother used hurtful and offensive comments to “encourage” them to study hard. This parenting approach may be linked to certain traditional cultural values such as Confucian and Taoist philosophies. Confucianism and Taoism are two prominent philosophical traditions in China that have shaped Chinese culture for centuries. Confucianism emphasizes hierarchical relationships, family obligations, and the importance of education. Taoism emphasizes the concept of dualism, highlighting the cyclical nature of existence and the interplay between opposing forces or extremes. This nuanced intermingling of philosophies further augments the intricacies underpinning parental approaches and their endeavours to “encourage” their children in traversing the labyrinthine pathways of life’s challenges. That is, these cultural values may contribute to a parenting style characterized by authoritarian tendencies, where Asian parents often expect their children to strive for academic success and pursue “saintly” occupations (Chen et al., 2021; Huang et al., 2017). While some studies have demonstrated the positive outcomes of Asian authoritarian parenting, such as enhanced emotional intelligence (Huang et al., 2017), and academic performance (Chen et al., 2021), our study highlights a hypothesis related to the impact of this parenting style on individuals with SMI. The authoritative parenting approach, which places high expectations and emphasizes criticism for failures or inadequacies, may create additional expectations and stress for individuals whose capacity and capability are limited by their mental illness. This could potentially weaken their sense of worth and inhibit their motivational functioning. It is important to note that the influence of culture on identity development in the context of mental illness is complex and multifaceted. By considering the cultural influences, we can better understand the unique challenges faced by Chinese adolescents with SMI and the potential impact of cultural values on their experiences.

According to the proposed model of “The dynamic interactions of Chinese adolescents’ identity and mental illness”, the current study suggests that early identity issue has a cumulative influence on one’s development, but individual subjectivity is an important source of negative identity and enduring identity, which can modify the cumulative process of early crises throughout the life course. Adolescents in this study also perceived that their personality characteristics and values and beliefs, provided a chance for adolescents choosing to adopt a negative value. Associated with biological features, it weakened their attempts to develop a positive identity, made them prone to develop a negative one. Although the literature did not explore the identity formation process from a multidimensional perspective, few recent clinical studies on individuality and identity formation support the finding of the importance of individual subjectivity and characteristics (McAneney et al., 2015; Milić et al., 2019; Pfeifer & Berkman, 2018).

This study further finds that perceived negative identity in relation to accumulated persistent stresses and ineffective coping strategies could be prolongated and aggravated by multiple factors across the life span, and continue into later adolescence. Although negative statuses of identity and the experience of mental illness symptoms were studied by researchers when it is more visible in the formation stage, the issue might begin at a much earlier time than most researchers and professionals realize. A longitudinal study conducted by Fernandez-Mendoza et al. (2021), reflects one finding in this study, that early-onset SMI symptoms in childhood, (typically insomnia), accumulate and persist over time, is an important factor in young people’s mental illness. Further support for this study can be found in recent research on problematic and pathological identity, which found that young people with more psychological problems displayed a more troublesome personality and identity (McLean et al., 2020).

In addition to the concept of negative identity, this study offers an adolescent perspective and proposes a new identity status, Buddha-like numbness, whereas numbness in the midst of morbidity is similar to the concept of identity diffusion (Marcia, 1966). Consistently with other literature, the study found that the illness experiences closely related to one’s identity formation (Chiba et al., 2020; Greenwood, 2020), and that this negative identity status was based on a personal value system associated with the personal reflexivity of the illness experience, the pessimistic evaluation of reality and subjective empathy. From the dynamic perspective of self and identity, this study explores the factors and processes of adolescents’ identity formation; it also recognizes the role adolescents themselves play in predisposing the sense of negative self and identity and mental illness symptoms, and in promoting the prolongation and severity of identity issue in the interactive context. The interactive effects within systems are also subject to perceived person-specific characteristics which are overlooked by the ecological theories (Bronfenbrenner, 1979). These include but are not limited to values, beliefs and personality traits.

In contrast to most existing research on identity reconstructions in relation to chronic or severe illnesses (Furr, 2022; Ho & Goh, 2017; Jaiswal et al., 2020; Kerr et al., 2019; Oris et al., 2018), this study reveals that adolescents’ illness identity is a fluctuating and non-linear process. However, despite these fluctuations, their reconstructed illness identity tends to be predominantly negative. This negativity is strongly influenced by the profound sense of stigma experienced by adolescents during the early stages of their illness. Stigma refers to society’s negative attitudes, beliefs, and perceptions towards individuals with specific conditions or attributes. Stigma significantly influences their understanding and acceptance of their illness, leading to negative self-perception and illness-related shame. This negative view becomes integrated into their overall identity, shaping how they perceive themselves and their position in the world. Consequently, the intense shame experienced during the early stages perpetuates the negative aspects of their self-concept, forming an enduring illness-centric negative self-image. That is, even in the later stages when adolescents with SMI may accept their condition, the negative self-perception lingers. This can be explained from a life course viewpoint. The life course perspective emphasizes the long-term effects of early experiences and developmental trajectories on individual’s lives. In the case of adolescents with SMI, the early experience of stigma and shame surrounding their illness has a lasting impact on their sense of self and identity. This identification with the negative aspects hinders their ability to develop a positive sense of self and may impact their social interactions, aspirations, and overall psychological well-being.

## Conclusions

Our results provide useful suggestions for improving school-based mental health work and service. For example, a positive intervention on self and identity can be applied together with other interventions for adolescents. According to the adolescents participated in this study, school staffs need to pay attention to peer relationships of adolescents with SMI and help them rebuild and remain healthy peer relationships. In addition, the professional rehabilitation support and service should be also a concern for schools. Schools are responsible to work with psychological and mental health professionals such as educational psychologists, and/or clinical psychologists. Psychologists have more professional experiences than teachers, and their knowledge and experiences can be used not only to support the well-being and mental health of adolescents, but also to provide professional advice for adolescents with SMI in their educational studies, and future plans after they return to school, to help adolescents with SMI deal with developmental challenges and personal stresses more effectively. In a longer term, it can potentially help adolescents establish a stable and positive self and identity.

Nevertheless, this study was limited by research limitations and academic pressures. First, because only one round of interview was conducted with participants, the results highlighted were only relevant to a narrow range of self and identity formation and did not allow for the identification of significant differences in negative identities due to different types of SMI and gender. With more time and monetary resources, future researchers could examine the study topic in more depth and find more detailed and meaningful results through research. This study was conducted based on the voices of adolescents with SMI in China. Therefore, the results of this study may not be applicable to other countries or regions. However, despite the cultural and situational specificity of the context of this study, the globalization trend has brought about quite common characteristics to the environment in which child and adolescents grow up.

## Data Availability

The data and materials that support the findings of this study are available from the authors upon reasonable request.
